# PTPN9 regulates HER3 phosphorylation during trastuzumab treatment and loss of PTPN9 is a potential biomarker for trastuzumab resistance in HER2 positive breast cancer

**DOI:** 10.1002/cac2.12632

**Published:** 2024-11-24

**Authors:** Abul Azad, Maryam Arshad, Daniele Generali, Katharina Feldinger, Merel Gijsen, Carla Strina, Mariarosa Cappelletti, Daniele Andreis, Russell Leek, Syed Haider, Pirkko‐Liisa Kellokumpu‐Lehtinen, Ioannis Roxanis, Adrian Llewellyn Harris, Abeer Mahmoud Shaaban, Heikki Joensuu, Anthony Kong

**Affiliations:** ^1^ Department of Oncology The Weatherall Institute of Molecular Medicine University of Oxford Oxford UK; ^2^ Institute of Genomic and Cancer Sciences Vincent Drive, University of Birmingham Birmingham UK; ^3^ Comprehensive Cancer Centre Kings’ College London London UK; ^4^ U.O. Multidisciplinare di Patologia Mammaria U.S Terapia Molecolare e Farmacogenomica A.O. Instituti Ospitalieri di Cremona Viale Concordia 1 Cremona Italy; ^5^ Biostatistics and Clinical Trials Unit Istituto Scientifico Romagnolo per lo Studio e la Cura dei Tumori IRCCS, Forlì‐Cesena Meldola Italy; ^6^ The Institute of Cancer Research London UK; ^7^ Department of Oncology Tampere University Hospital and Tampere University Tampere Finland; ^8^ Department of Cellular Pathology Oxford University Hospitals and Oxford Biomedical Research Centre Oxford UK; ^9^ Department of histopathology Queen Elizabeth Hospital Birmingham Birmingham UK; ^10^ Department of Oncology Helsinki University Hospital and University of Helsinki Helsinki Finland

**Abbreviations**
ADAM10/17: a disintegrin and metalloproteinase 10/17
EGFR: epidermal growth factor receptor
HER2: human epidermal growth factor receptor 2
HER3: human epidermal growth factor receptor 3
IRS: immunoreactive scoring system
PDOs: patient‐derived organoids
T‐DM1: ado‐trastuzumab emtansine
T‐Dxd: trastuzumab deruxtecan
TMA: tissue microarray, PTPN9, tyrosine‐protein phosphatase non‐receptor type 9

Although trastuzumab does not bind to human epidermal growth factor receptor 3 (HER3), it dephosphorylates HER3 through a previously unknown mechanism. In addition, HER3 is reactivated during prolonged trastuzumab treatment and upon resistance [1]. Previous study showed that tyrosine‐protein phosphatase non‐receptor type 9 (PTPN9) inhibits STAT3/STAT5 signalling by dephosphorylation of epidermal growth factor receptor (EGFR) and human epidermal growth factor receptor 2 (HER2) in breast cancer [2], but how this would affect HER3 was not analyzed especially in relation to trastuzumab treatment. We investigated the role of PTPN9 in HER3 signaling in relation to trastuzumab treatment and resistance in HER2 positive breast cancer. The materials and methods applied in this research were described in the supplementary materials.

We showed that PTPN9 was upregulated after trastuzumab treatment in both SKBR3 and BT474 cells (Figure 1A), but this is not the case for two other PTPs which are known to regulate EGFR and HER2 respectively, PTP1B and PTPN13 [3, 4] (Supplementary Figure S1A). The upregulation of PTPN9 occurred concomitantly with a decrease in the phosphorylation of HER3 and its downstream effector protein kinase B (PKB or Akt), but not HER2 and EGFR (Figure 1A and Supplementary Figure S1B). Moreover, HER3 and Akt were reactivated in trastuzumab‐resistant SKBR3 and BT474 cells with a concomitant decreased PTPN9 expression. In contrast, EGFR and HER2 phosphorylation was not decreased by trastuzumab treatment but was further increased during trastuzumab resistance, which was previously shown to be due to a disintegrin and metalloproteinase 10/17 (ADAM10/17) mediated HER ligand activation [1, 5]. In immunofluorescence studies, PTPN9 expression was upregulated in cytoplasm and co‐localized with the cytoplasmic HER3 following trastuzumab treatment for 4 hours in both SKBR3 and BT474 cells (Supplementary Figure S1C), correlated with a decrease of pHER3 seen in the western blot at this time point. PTPN9 expression was decreased again in trastuzumab‐resistant BT474 and SKBR3 cells (Supplementary Figure S1C) which was correlated with a reactivation of HER3. Similarly, PTPN9 expression and pHER3 levels were seen to be inversely correlated during trastuzumab treatment in MDA‐MB‐453 and MDA‐MB‐361 cells (Supplementary Figure S1D). In relation to other anti‐HER2 therapies, trastuzumab and ado‐trastuzumab emtansine (T‐DM1) (and to much lesser extent for trastuzumab deruxtecan [TDxd] but not neratinib and pertuzumab monotherapy) could increase PTPN9 expression (Supplementary Figure S1E), although decreased HER3 and Akt phosphorylation was seen in all drugs, which may reflect the different mechanisms of action of these drugs. The trastuzumab‐based combination treatment also upregulated PTPN9 expression with concomitant decrease in HER3 and Akt phosphorylation (Supplementary Figure S1E).

**FIGURE 1:**
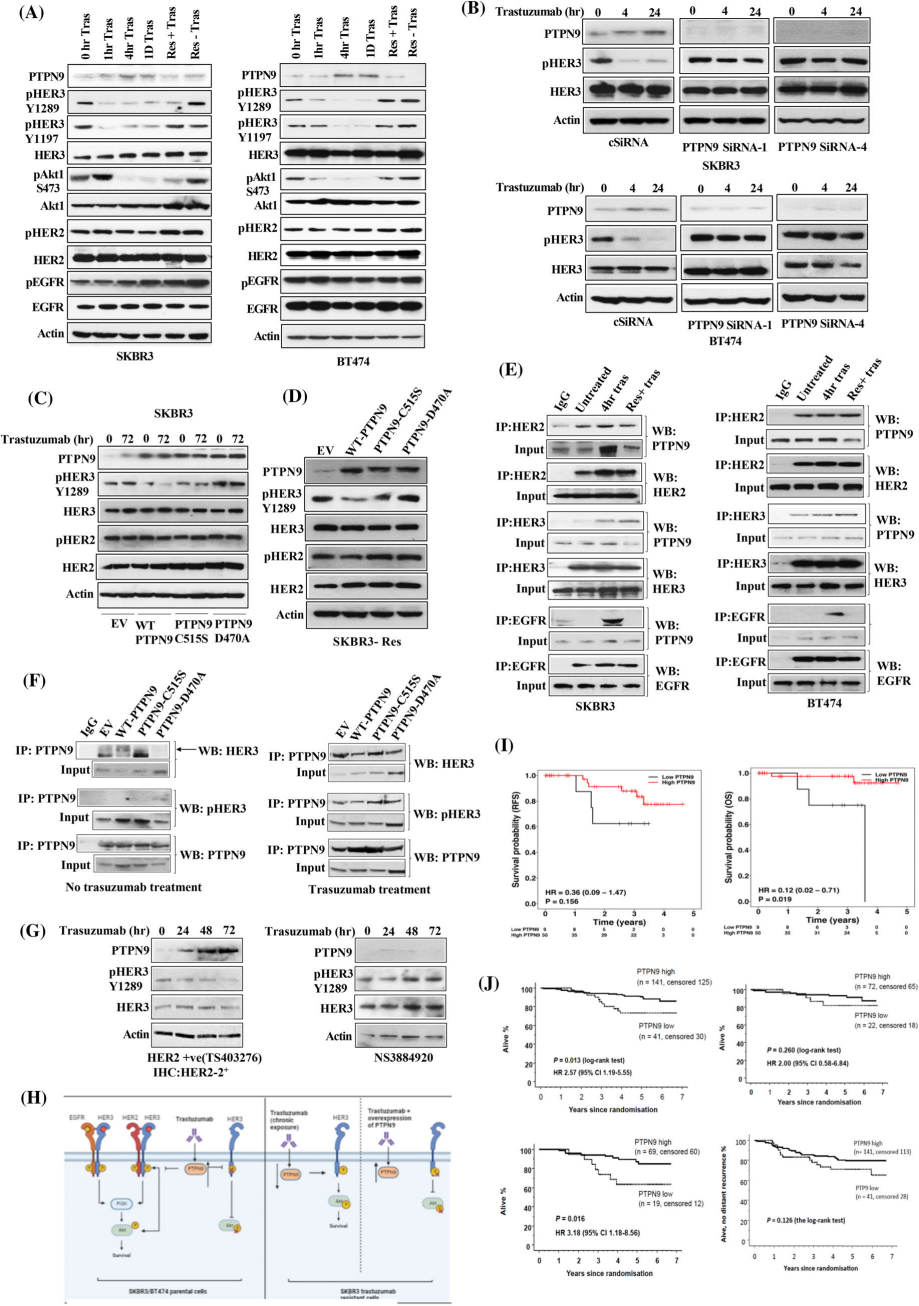
Trastuzumab upregulates PTPN9 which is associated with HER3 dephosphorylation and PTPN9 is a potential prognostic biomarker in HER2 positive breast cancer patients. (A) Both SKBR3 and BT474 cells were treated with 40 µg/mL of trastuzumab for indicated times and were assayed for expression and phosphorylation of indicated proteins by western blot. Immunoblots were also performed for indicated proteins from both trastuzumab‐resistant SKBR3 and BT474 cells and after withdrawal of trastuzumab from resistant cells for 24 hours. Actin represents equal loading control. (B) SKBR3 and BT474 cells were transfected with 50 nmol/L of control siRNA (csiRNA) and two independent specific siRNA against PTPN9. After a total of 48 hours’ transfection, cells were treated with trastuzumab for 4 and 24 hours. (C) SKBR3 cells were transfected with wild type (WT) and mutant PTPN9 overexpressing plasmids. Following transfection and selection with G418, the cells were seeded and treated with trastuzumab for 72 hours. The cells were lysed and analyzed for expression of PTPN9 and level of HER3 and HER2 phosphorylation. (D) Similarly, trastuzumab‐resistant SKBR3 cells were also transfected with WT and mutant PTPN9 overexpressing plasmids and were analyzed for expression of indicated proteins. In all cases, actin was used as loading control. (E) Interaction studies between PTPN9 and HER family receptors were performed in SKBR3 and BT474 cells ± trastuzumab for 4 hours and also in trastuzumab‐resistant SKBR3 and BT474 cells. Immunoprecipitations was performed with anti‐HER3, anti‐HER2, or anti‐EGFR antibodies before blotted for PTPN9 (top panels). In each case, immunoprecipitation was also blotted for the respective antibody to show equal immunoprecipitation was performed. IgG was used as a IP control. 5% of total extracts were used as input. (F) Immunoprecipitations was performed with anti‐PTPN9 antibody using cell lysates from SKBR3 cells transfected with empty vector (EV), WT PTPN9, mutant C515S and D470A forms of PTPN9 and were blotted for anti‐HER3 or anti‐pHER3 antibody. Immunoprecipitation was also done with the same antibody to show that equal immunoprecipitation was performed. IgG used as an IP control. 10% of total extracts was used as input. (G) Patient‐derived organoids (PDOs) from HER2 ve+ (left), normal tissue (right) were treated 40 ug/mL of trastuzumab for indicated times. Expression of indicated proteins were analysed by immunoblot. (H) Schematic illustrations of the regulation of HER3 signalling by PTPN9 in relation to trastuzumab treatment in HER2 positive breast cancer cells: in the parental SKBR3/BT474 cells, HER receptors dimerize with each other upon growth factor stimulation, resulting in the activation of HER3 and its downstream activator Akt, which leads to proliferation and survival. Following trastuzumab treatment in the parental SKBR3 cells, PTPN9 expression is upregulated. It interacts with EGFR, HER2 and HER3 although it only dephosphorylates HER3, leading to a decrease in Akt phosphorylation. In chronic exposure to trastuzumab leading to its acquired resistance, PTPN9 expression is decreased again, resulting in the reactivation of HER3 and its downstream effector Akt. Following an overexpression of PTPN9 in trastuzumab‐resistant cells, HER3 could be dephosphorylated again leading to a decrease in Akt phosphorylation. (I) Using the established IRS scoring system, the breast cancer patients from a cohort in Oxford (n = 59) were split into two groups according to PTPN9 IRS scoring of either 4 (n = 50) and higher or below 4 (*n* = 9). PTPN9 expression was split into low and high expression and the relapse‐free survival (Left) and overall survival (Right) between the two groups was assessed by Log‐rank test. (J) Similarly, PTPN9 expression in relation to overall survival between the two groups was also assessed in all (Left upper panel), trastuzumab treated (Right upper panel) and non‐trastuzumab treated (Left lower panel) of HER2 positive patients in FinHER trial. PTPN9 expression in relation to distant recurrence‐free survival between the two groups was further assessed in HER2‐positive FinHER patients. (Right lower panel) PTPN9 expression was split into low and high expression and the time‐to‐distant recurrences between the two groups for all FinHER patients was assessed by Log‐rank test. Abbreviations: T‐DM1, ado‐trastuzumab emtansine; T‐Dxd, trastuzumab deruxtecan; PDOs, patient‐derived organoids; IRS, immunoreactive scoring system; TMA, tissue microarray, PTPN9, tyrosine‐protein phosphatase non‐receptor type 9; EGFR, epidermal growth factor receptor; HER2, human epidermal growth factor receptor 2; HER3, human epidermal growth factor receptor 3; ADAM10/17, a disintegrin and metalloproteinase 10/17.

Next, we showed that PTPN9 knockdown using two independent siRNAs counteracted the decreasing effect of trastuzumab on HER3 phosphorylation in both SKBR3 and BT474 cells following optimization (Figure 1B and Supplementary Figure S2A‐B). PTPN9 knockdown also decreased the anti‐proliferative effects of trastuzumab in SKBR3 cells (Supplementary Figure S2C). Using two PTPN9‐mutants (catalytically inactive PTPN9‐C515S and substrate trapping mutant PTPN9‐D470A) [6], we showed that while overexpression of wild‐type PTPN9 led to a decrease of pHER3, this was not the case for PTPN9‐D470A and catalytically inactive PTPN9‐C515S in the parental (both untreated and trastuzumab treated) and resistant SKBR3 cells (Figure 1C‐D). Although HER2 phosphorylation was slightly decreased with the overexpression of wild‐type PTPN9 in the resistant cells (Figure 1D), the activation of EGFR (Supplementary Figure S2D) was not affected by PTPN9 over‐expression.

We further investigated the interaction of PTPN9 with HER receptors and found that there was an interaction of PTPN9 with HER2 at baseline with an increased interaction of PTPN9 with EGFR, HER2 and HER3 in the parental SKBR3 and BT474 cells when treated with trastuzumab (Figure 1E and Supplementary Figure S2E). Upon trastuzumab acquired resistance, the interaction of PTPN9 with EGFR but not with HER2 or HER3 (Figure 1E and Supplementary Figure S2E) was decreased. This suggests that although PTPN9 interacts with EGFR, HER2 and HER3 (or their dimers ± complex) during trastuzumab treatment, EGFR plays an important role in HER3 dephosphorylation by recruiting PTPN9. However, EGFR, HER2 and HER3 knockdown all led to a decrease in trastuzumab‐induced PTPN9 upregulation (Supplementary Figure S2F), indicating all three receptors are involved in upregulating PTPN9 following trastuzumab treatment although the exact mechanisms could be complex and beyond the scope of this manuscript. We found that compared to empty vector, WT PTPN9 directly interacted with HER3 at the basal level whereas this interaction was decreased in catalytically inactive mutant C515S and was further reduced in the substrate trapping mutant D470A (Figure 1F). However, reblotting of the same membrane showed increased pHER3 in the substrate trapping mutant D470A at baseline as result of dominant negative mutant. Upon trastuzumab treatment, there was also increased pHER3 in D470A whereas pHER3 was decreased in the WT PTPN9. Thus, our results indicated that HER3 may be a direct substrate of PTPN9.

To ensure clinical relevance, further experiments were performed in patient‐derived organoids (PDOs) with different HER2 status, which were previously generated [7]. We found that PTPN9 was upregulated following trastuzumab treatment with a concomitant decrease in HER3 phosphorylation in a HER2+ve (IHC2+ and FISH+ve) PDO line TS403276 (Figure 1G), which was previously shown to recapitulate the tumour characteristics of the parental tumour [7]. However, the inverse relation between pHER3 and PTPN9 could also be seen to a lesser extent in the control PDO derived from normal breast tissue (Figure 1G) as well as a HER2‐low PDO line and a HER2 negative breast cancer PDO line (Supplementary Figure S2G‐H), which were previously shown to be insensitive to trastuzumab [7]. The proposed model of a novel interaction between PTPN9 and HER3 during trastuzumab treatment is depicted in Figure 1H.

Next, we assessed PTPN9 levels in HER2 positive breast cancer patients who underwent a window study [5, 8] after IHC PTPN9 staining was optimised in BT474 cell pellets (Supplementary Figure S3A). We showed that PTPN9 levels were significantly decreased at day 21 after one dose of trastuzumab monotherapy compared to baseline (Supplementary Figure S3B, *P* = 0.03) although the 24‐hour post‐trastuzumab treatment biopsy samples were not available for comparison. However, after neoadjuvant docetaxel chemotherapy with trastuzumab when most tumours had responded [5, 8], there was no difference in PTPN9 levels (Supplementary Figure S3C). The pre‐treatment PTPN9 levels correlated with clinical response (post/pre‐treatment tumour size) after one dose of trastuzumab at day 21 (R^2^ = 0.29, *P* = 0.047) (Supplementary Figure S3D) and there was a greater decrease of tumour size for patients with higher PTPN9 levels. However, there was no correlation between baseline PTPN9 levels and changes in tumour size at definitive surgery after neoadjuvant docetaxel chemotherapy and trastuzumab treatment (data not shown).

Using the established immunoreactive (IRS) scoring system [9] in the tissue microarrays (TMAs) of HER2 positive breast tumours stained for PTPN9 expression (Supplementary Figure S3E), samples were grouped into low PTPN9 (score < 4) or high PTPN9 (IRS ≥ 4). The patient and tumour characteristics stratified by PTPN9 expression are shown in Supplementary Table S1. There were no statistically significant differences in tumour size, nodal status, ER status, grade of tumours, or age of patients between patients with low PTPN9 and high PTPN9 expression (Supplementary Table S1). Patients with low PTPN9 levels had a trend towards a poorer relapse‐free survival (RFS) compared to patients with high PTPN9 (HR = 0.36, 95% CI = 0.09‐1.47, *P* = 0.156) although this was not statistically significant (Figure 1I, left panel). However, patients with low PTPN9 had a poorer overall survival (OS), compared to high PTPN9 (HR = 0.12, 95% CI = 0.02‐0.71, *P* = 0.019) (Figure 1I, right panel), which was also confirmed in a multivariate analysis (Supplementary Table S2). We also assessed PTPN9 expression in breast tumours from the FinHER trial [10] and showed that low PTPN9 levels were significantly associated with a poorer OS (HR = 2.57, 95% CI = 1.19‐5.55, *P* = 0.013) in all patients (Figure 1J, upper left panel). When further analyzing the subgroups according to whether they were treated with adjuvant trastuzumab or not, low PTPN9 levels was significantly associated with a poorer OS in non trastuzumab‐treated patients (HR = 3.18, 95% CI = 1.18‐8.56, *P* = 0.016) but not trastuzumab‐treated patients (HR = 2.00, 95% CI = 0.58‐6.84, *P* = 0.260) (Figure 1J). In addition, there was a trend (*P* = 0.126) for low PTPN9 group to have a shorter time to distant recurrence in all patients (Figure 1J). Similar trends were seen in both trastuzumab‐treated and non trastuzumab‐treated subgroups but they were also not statistically significant (data not shown).

In conclusion, our results demonstrated that PTPN9 was inversely correlated with HER3 phosphorylation during trastuzumab treatment and upon trastuzumab resistance, implicating its role in regulating HER3 dephosphorylation. It may also be a novel predictive and prognostic biomarker in HER2 positive breast cancer patients. More investigation would be required to validate PTPN9 as a predictive biomarker for targeted therapies in various cancers and as a potential target by allosteric inhibitor to reverse drug resistance in cancer treatments.

## AUTHOR CONTRIBUTIONS

Conceptualization Anthony Kong and Abul Azad. Abul Azad design experimental strategy, obtained and analyse data. Maryam Arshad and Abeer Mahmoud Shaaban helped to generate patient‐derived organoids. Abul Azad and Anthony Kong finalised the manuscript. All authors contributing to editing the manuscript and approved the final version.

## CONFLICT OF INTEREST STATEMENT

The authors declare no competing financial interests but the following competing non‐financial interests: Dr. Anthony Kong filed a patent of PTPN9 as a biomarker in relation to cancer treatment (international patent application No. PCT/GB2013/050057). However, this patent was not subsequently being maintained by further payments. All other authors declare no competing financial or non‐financial interests.

## FUNDING INFORMATION

Dr. Anthony Kong and Dr. Abul Azad were supported by Breakthrough Breast cancer (Grant number: CSF‐Kong) through Holbeck Charitable Trust. We would also like to acknowledge the funding from University of Birmingham ECMC (Experimental Cancer medicine Centres) and MRC proximity to Discovery award for part of the work. Prof A Shaaban is supported by Birmingham CRUK Centre grant.

## ETHICS APPROVAL AND CONSENT TO PARTICIPATE

To generate patient‐derived organoids, breast cancer tumours and normal tissues were obtained after surgical resection from Queen Elizabeth Hospital, Birmingham in the UK (under the ethics of University of Birmingham Human Biomaterials Resource Centre [HBRC] reference 16‐259). The trastuzumab window study was conducted at UOM Patologia Mammaria‐Az. Instituti Ospitalieri di Cremona with appropriate local ethical approval (Protocol CE‐21392/2012). The TMA slides from a cohort of HER2 positive breast cancer patients were provided by Oxford Radcliffe Biobank after an internal application and reviewed by the Scientific and Ethical Review Committee. The use of these TMA slides complies with the Human Tissue Act 2004 (UK). The use of FinHER trial samples for this study was done under the study proposal approved by the Helsinki University Central Hospital Ethics Committee (331/E6/07, 17 Oct 2007). The trial patients provided written informed consent for the use of their tumour tissue material for the FinHER trial‐related research.

## Supporting information



Supporting Information

## Data Availability

All the original western blots and raw data are available from the authors upon request.
