# Supplementary material for: PTPN9 regulates HER3 phosphorylation during trastuzumab treatment and loss of PTPN9 is a potential biomarker for trastuzumab resistance in HER2 positive breast cancer
Source: Cancer Commun (Lond). 2024 Nov 24;45(1):68–73. doi: 10.1002/cac2.12632 (PMC11758155; doi:10.1002/cac2.12632)
Supplement: Supplementary file 1 — Supporting Information [file CAC2-45-68-s001.docx]

**Supplementary Materials**

**PTPN9 regulates HER3 phosphorylation during trastuzumab treatment and loss of PTPN9 is a potential biomarker for trastuzumab resistance in HER2 positive breast cancer**

Abul Azad^1,2,3^, Maryam Arshad^2,3^, Daniele Generali^4^, Katharina Feldinger^1^, Merel Gijsen^1^, Carla Strina^4^, Mariarosa Cappelletti^4^, Daniele Andreis^4,5^, Russell Leek^1^, Syed Haider^1,6^, Pirkko-Liisa Kellokumpu-Lehtinen^7^, Ioannis Roxanis^6,8^, Adrian Llewellyn Harris^1^, Abeer Mahmoud Shaaban^9^ Heikki Joensuu^10^, Anthony Kong^1,2,3,*^

1. Department of Oncology, The Weatherall Institute of Molecular Medicine, University of Oxford, Oxford, OX3 9DS, United Kingdom.
2. Institute of Genomic and Cancer Sciences, Vincent Drive, University of Birmingham, Birmingham, B15 2TT, United Kingdom.
3. Comprehensive Cancer Centre, Kings’ College London, London, SE1 1UL, United Kingdom.
4. U.O. Multidisciplinare di Patologia Mammaria, U.S Terapia Molecolare e Farmacogenomica, A.O. Instituti Ospitalieri di Cremona, Viale Concordia 1, 26100, Cremona, Italy.
5. Biostatistics and Clinical Trials Unit, Istituto Scientifico Romagnolo per lo Studio e la Cura dei Tumori IRCCS, Meldola (FC), Italy.
6. The Institute of Cancer Research, London, SW7 3RP, United Kingdom.
7. Department of Oncology, Tampere University Hospital and Tampere University, Tampere, 33520, Finland.
8. Department of Cellular Pathology, Oxford University Hospitals and Oxford Biomedical Research Centre, Oxford, OX3 7JX, United Kingdom.
9. Department of histopathology, Queen Elizabeth Hospital Birmingham, Birmingham, B15 2WB, United Kingdom.
10. Department of Oncology, Helsinki University Hospital and University of Helsinki, Helsinki, 00260, Finland.

*Corresponding author:

Anthony Kong, King’s College London, Guy’s Campus, New Hunt’s House, London, SE1 1UL,

United Kingdom, E-mail: [anthony.kong@kcl.ac.uk](mailto:anthony.kong@kcl.ac.uk).

**Supplementary Table S1. Patient and tumour characteristics stratified by PTPN9 expression.**

| **Characteristic** | | **Total cases** | PTPN9 expression | | ***P* value** |
| --- | --- | --- | --- | --- | --- |
|  |  |  | IRS score < 4 | IRS score ≥ 4 |  |
| **Tumour size, cm** |  |  |  |  | 0.140 |
|  | **< 2 cm** | 18 | 5 (56%) | 13 (26%) |  |
|  | **2-5 cm** | 35 | 3 (33%) | 32 (64%) |  |
|  | **≥ 5 cm** | 6 | 1 (11%) | 5 (10%) |  |
| **Nodal status** |  |  |  |  | 1.000 |
|  | Negative | 33 | 5 (56%) | 28 (57%) |  |
|  | Positive | 25 | 4 (44%) | 21 (43%) |  |
|  | Unknown | 1 |  |  |  |
| **ER status** |  |  |  |  | 1.000 |
|  | Negative | 15 | 2 (22%) | 13 (27%) |  |
|  | Positive | 43 | 7 (78%) | 36 (73%) |  |
|  | Unknown | 1 |  |  |  |
| **Grade** |  |  |  |  | 0.236 |
|  | 3 | 43 | 5 (56%) | 38 (76%) |  |
|  | 1-2 | 16 | 4 (44%) | 12 (24%) |  |
| **Age** |  |  |  |  | 1.000 |
|  | 55 or above | 41 | 6 (67%) | 35 (70%) |  |
|  | Under 55 | 18 | 3 (33%) | 15 (30%) |  |

**Supplementary Table S2. Multivariate analysis assessing the independence of PTPN9 from clinical co-factors as a prognostic value for RFS and OS.**

| **Survivals** | **Coef** | **Exp (coef)** | **Se (coef)** | **Z value** | ***P* value** |
| --- | --- | --- | --- | --- | --- |
| **RFS^a^** | | | | | |
| Group | **-2.1** | **0.12** | **1.13** | **-1.86** | **0.06** |
| Age | 1.67 | 5.3 | 1.15 | 1.46 | 0.14 |
| Grade | -0.06 | 0.94 | 0.99 | -0.06 | 0.95 |
| Nodes | -1.20 | 0.30 | 1.01 | -1.19 | 0.23 |
| ER | 1.17 | 3.24 | 0.78 | 1.52 | 0.13 |
| Factor (size) 2 | 0.98 | 2.67 | 1.28 | 0.77 | 0.44 |
| Factor (size) 3 | 1.79 | 6.00 | 1.48 | 1.21 | 0.23 |
| Likelihood ratio test=11.7 on 7 df, p=0.11 n= 57, number of events= 9  (2 observations deleted due to missing data) | | | | | |
| **OS^a^** | | | | | |
| Group | **-4.67** | **0.01** | **2.00** | **-2.33** | **0.02** |
| Age | 3.70 | 40.53 | 2.52 | 1.47 | 0.14 |
| Grade | -0.09 | 0.91 | 1.61 | -0.06 | 0.95 |
| Nodes | -3.37 | 0.03 | 2.59 | -1.30 | 0.19 |
| ER | 1.53 | 4.64 | 1.59 | 0.97 | 0.33 |
| Factor (size) 2 | 0.14 | 1.15 | 2.55 | 0.056 | 0.96 |
| Factor (size) 3 | 1.12 | 3.08 | 2.73 | 0.41 | 0.68 |
| Likelihood ratio test=15.1 on 7 df, p=0.0353 n= 57, number of events= 5 (2 observations deleted due to missing data) | | | | | |

**^a^**Coxph (formula = survobj ~ Group + Age + Grade + Nodes + ER + factor (Size), data = ann.dataRFS = Relapse Free Survival; OS = Overall Survival.

**Supplementary figure legends**


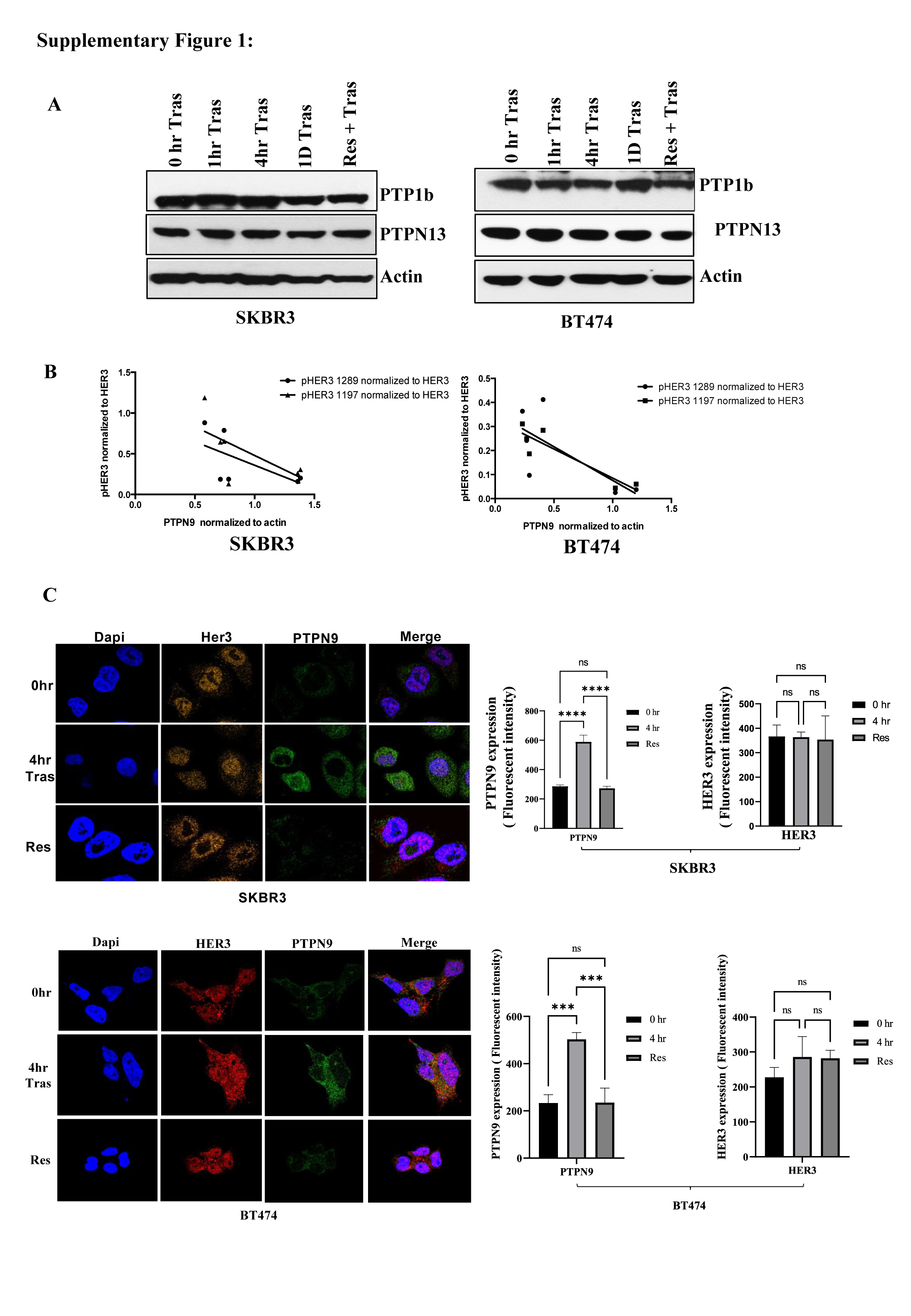


**Supplementary Figure S1**. **Trastuzumab upregulates PTPN9 which is associated with HER3 dephosphorylation.**

1. Both parental and trastuzumab-resistant SKBR3 and BT474 cells were treated with 40 µg/ml of trastuzumab for indicated times and analysed for expression of PTP1b and PTPN13. for indicated proteins. Actin is used as loading control.
2. The quantification of the representative blots shown in Figure 1A was done; the intensity of pHER3_1289_ and pHER3_1197_ in relation to HER3 was correlated with the intensity of PTPN9 blots in relation to actin.
3. Left: localization of PTPN9 and HER3 in the parental SKBR3 and BT474 cells following treatment with trastuzumab for 4 hours and also in trastuzumab-resistant SKBR3 and BT474 cells continuously treated with trastuzumab. Representative images of three independent repeats were captured by confocal microscopy. Following treatment, cells were fixed and stained for PTPN9 and HER3. DNA was localized by DAPI. Right: quantification of cells with PTPN9 and HER3 staining from multiple images of randomly selected fields. 50-70 cells were examined from different fields of repeated experiments.
4. Both MDA-MB-453 and MDA-MB-361 cells were treated with 40 µg/ml trastuzumab for indicated time and expression of protein level were analysed as indicated.

(E) Left: SKBR3 cells were treated with 40 ug/ml of trastuzumab, 10ng /ml of neratinib, 1 ug/ml of T-DM1, 10 ug/ml of T-DXd either alone or their combination for 24 hrs before being analyzed for expression of indicated proteins. Right: SKBR3 cells were also treated with 40 ug/ml of trastuzumab, 20 µg/ml of pertuzumab, 10 ng /ml of neratinib either alone or their combinations for 24 hrs before being analysed for indicated protein expression. Actin was used as a loading control.


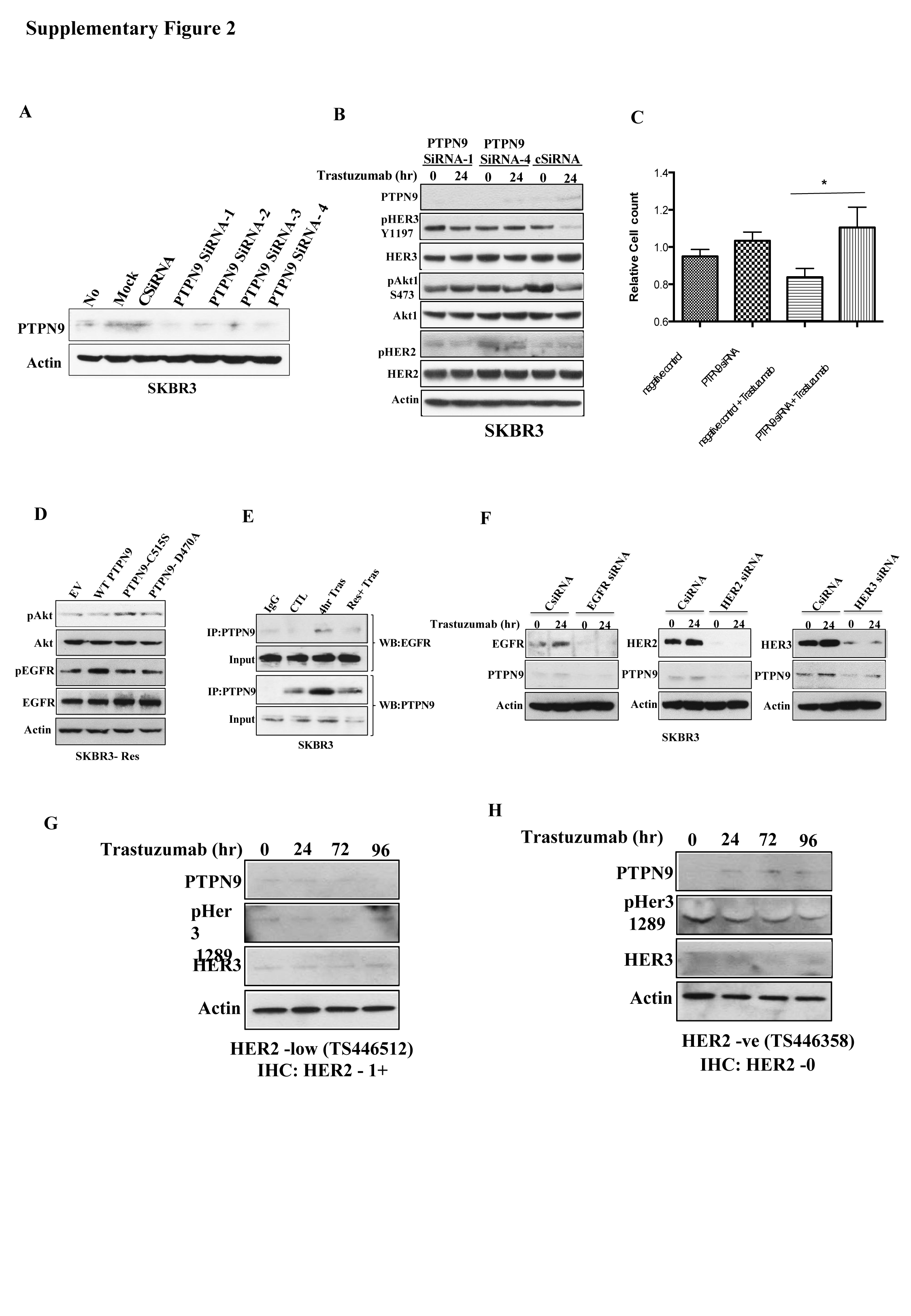


**Supplementary Figure S2.** **Loss of PTPN9 is associated with trastuzumab resistance**

1. PTPN9 knockdown was verified by western blot after transfection of SKBR3 cells with four different specific siRNA against PTPN9. The cells were also mock treated (transfection reagent only) or transfected with control non-specific siRNA with actin as the loading control.
2. SKBR3 cells were transfected with control and PTPN9 siRNA as described above for 48 hrs, which was followed by trastuzumab treatment for 24 hrs. Cells lysates were prepared and were analyzed for protein expression as indicated.
3. The parental SKBR3 cells were transfected with either a control siRNA or siRNA against PTPN9. They were plated and treated for 3 days with 40ug/ml trastuzumab and the remaining cells wLoere counted using a cell counter.
4. Trastuzumab resistant SKBR3 cells containing WT and mutant PTPN9 overexpressing plasmids were analysed for expression of indicated proteins. In all cases, actin was used as loading control.
5. Immunoprecipitation was performed with PTPN9 to analyse the interaction between EGFR in SKBR3 parental cells following trastuzumab treatment for 4 hrs and SKBR3 trastuzumab resistant cells. Input is 5% of total extract used in the immunoprecipitation.
6. EGFR, HER2 and HER3 were specifically knocked down in SKBR3 cells. Following treatment with trastuzumab for 24 hours and after a total of 48 hours of transfection, the cells were lysed and analyzed for indicated proteins.

(G-H) Patient-derived organoids from HER2-low and HER2-ve tumours were treated 40 ug/ml of Trastuzumab for indicated times. Expression of indicated proteins were analyzed by immunoblot.

**Supplementary Figure S3.** **Pre-treatment PTPN9 levels correlated with clinical response**

1. Cell pellets from BT474 cells transfected with either a negative control siRNA or siRNA against PTPN9 were stained for PTPN9 to ensure antibody specificity.
2. HER2 positive breast cancer patients were given one dose of trastuzumab (8mg/kg) followed by 4 cycles of neoadjuvant docetaxel chemotherapy 100mg/m^2^ q21 with 6mg/kg trastuzumab before surgery. Paired tissue samples (pre- and post-treatment) at day 21 after trastuzumab (8mg/kg) monotherapy window study, were stained for PTPN9 expression.
3. Paired tissue samples (pre- and post-treatment) after neoadjuvant chemotherapy and trastuzumab were stained for PTPN9 expression.
4. Basal PTPN9 levels were correlated with clinical response (post/pre-treatment tumour size) at day 21. Scatter plots showing the relationship between the post/pre-treatment tumour size and basal PTPN9 levels and their relationships were examined using Spearman-Rank correlation.

(E) Examples of PTPN9 IHC staining in TMA from HER2 positive breast cancer patients.

**Additional supplementary information (not included in the Letter to the Journal)**

**Background**

The ErbB family of receptor protein-tyrosine kinase (RTK/PTK) consists of four members EGFR/ErbB1, HER2/ErbB2, HER3/ErbB3 and HER4/ErbB4 [[11](#_ENREF_11)]. Overexpression or amplification of HER2 is responsible for around one fifth of breast cancers and is associated with poor prognosis [[12-14](#_ENREF_12)]. Trastuzumab, a humanized monoclonal antibody, has changed the treatment paradigm of patients with HER2 overexpressing breast cancer in the neoadjuvant, adjuvant and metastatic settings [[15](#_ENREF_15)]. However, some patients do not respond to trastuzumab treatment at all (*de novo* resistance) and initially responsive patients usually develop acquired resistance to trastuzumab within one year of treatment [[16](#_ENREF_16), [17](#_ENREF_17)]. Since trastuzumab approval, multiple anti-HER2 treatments are now approved in the treatment of early and advanced HER2 positive breast cancers. However, trastuzumab remains the first-line agent either as a combination with other agents or as antibody conjugates such as TDM1 and trastuzumab deruxtecan (T-DXd) [[16](#_ENREF_16)]. Thus, it still remains important to understand the resistance mechanisms of trastuzumab. Although various resistance mechanisms to trastuzumab have been extensively studied [[17](#_ENREF_17)], the precise mechanisms of acquired and *de novo* resistance are still poorly understood.

HER3 is frequently expressed in HER2 overexpressing breast cancers and was shown to be crucial for HER2-mediated signalling. Although HER3 lacks an active kinase domain, it is a potent activator of the PI3K/Akt signalling pathway when transphosphorylated by other receptors [[18](#_ENREF_18)]. HER3 is dephosphorylated within a few hours of trastuzumab and gefitinib treatment [[1](#_ENREF_1), [19](#_ENREF_19)], but the underlying mechanism is still unclear. Furthermore, HER3 is reactivated during prolonged gefitinib and trastuzumab treatment and upon resistance [[1](#_ENREF_1), [19](#_ENREF_19)]. Thus, indicating that HER3 may not only be important for tumorigenesis but also be driving drug resistance. This importance of HER3 dephosphorylation and reactivation in HER2 overexpressing breast cancer led us to investigate the mechanisms of HER3 regulation in order to develop strategies to overcome trastuzumab resistance.

Protein-tyrosine phosphatases (PTPs) are enzymes that antagonize the action of tyrosine kinases [[20](#_ENREF_20)]. The dephosphorylation of receptor tyrosine kinases by PTPs can inhibit oncogenic growth, decrease invasion/migration of cancer cells, control apoptosis and may play an important role in tumour suppression. Although the role of tyrosine kinases has been extensively studied, much less is known about the role of PTPs [[3](#_ENREF_3)]. PTP1B [[3](#_ENREF_3)] and PTPN6/Shp-1 [[21](#_ENREF_21)] can dephosphorylate EGFR. It was previously demonstrated that PTPN2 is a therapeutic target and a diagnostic biomarker in pancreatic cancer through targeting Stat1 and EGFR [[22](#_ENREF_22)]. PTPN13 negatively regulates HER2 activation in ovarian cancer and has been identified as a tumour suppressor in breast cancer [[4](#_ENREF_4)]. PTPN12 has been identified as a tumour suppressor in triple negative breast cancer (TNBC) and suppresses cell proliferation by interaction with and inhibition of multiple oncogenic tyrosine kinases including HER2 and EGFR [[23](#_ENREF_23)]. One study showed that PTPN9 inhibits STAT3/STAT5 signalling by dephosphorylation of EGFR and HER2 in breast cancer [[24](#_ENREF_24)] but how this would affect HER3 was not analysed. Although most of these phosphatases have been found to play a role in dephosphorylating EGFR/HER2, their role has not been implicated in relation to resistance to trastuzumab treatment. Moreover, the specific phosphatase that regulates HER3 phosphorylation in relation to trastuzumab treatment is yet unknown. The objective of our study was to investigate the role of PTPN9 in HER3 signalling in relation to trastuzumab treatment and resistance in HER2 positive breast cancer.

**Discussion**

Although trastuzumab binds to domain IV of HER2 and decreases Akt phosphorylation through PTEN activation [[9](#_ENREF_9)], the mechanism of how it dephosphorylates HER3 is unclear. In addition, HER3 is reactivated during prolonged treatments with trastuzumab and TKIs such as gefitinib [[1](#_ENREF_1), [19](#_ENREF_19), [25](#_ENREF_25)]. It was postulated that HER3 phosphorylation recovery could be an important mechanism by which breast cancer cells confer resistance to HER inhibitors and an unidentified protein tyrosine phosphatase (PTP) could be responsible for dephosphorylating HER3 upon acute TKI treatment [[19](#_ENREF_19)]. Here, we have uncovered a protein tyrosine phosphatase that regulates HER3 phosphorylation, which is involved in the resistance mechanism to various targeted therapies [[19](#_ENREF_19), 20, 21]. We have shown that the regulation of HER3 signalling is intimately linked with PTPN9 expression during trastuzumab treatment. Short-term trastuzumab treatment induced upregulation of PTPN9 level and HER3 dephosphorylation, but this was followed by a decreased PTPN9 level and HER3 reactivation upon acquired trastuzumab resistance. PTPN9 knockdown prevented trastuzumab from inducing HER3 dephosphorylation. In addition, overexpression of PTPN9 significantly reduced HER3 phosphorylation and prevented its reactivation upon prolonged trastuzumab treatment. It will be important to study the role of PTPN9 in regulating HER3 activation in a large panel of breast cell lines with varying HER2 expression and trastuzumab sensitivity to further assess its role in trastuzumab innate resistance. To ensure clinical relevance, it would also be important to investigate the correlation of PTPN9 expression with HER3 phosphorylation in a larger panel of patient-derived organoid models with varying HER2 status.

The proposed model of a novel interaction between PTPN9 and HER3 is depicted in Figure 1H. Trastuzumab treatment induces an upregulation of PTPN9, which interacts with EGFR, HER2 and HER3 but only HER3 was dephosphorylated.in the parental cells. However, upon long-term trastuzumab treatment and acquired resistance, PTPN9 expression was decreased but it continues to interact with HER2 and HER3 but interaction with EGFR is lost. Thus, a reduced level of PTPN9 and a decreased interaction of PTPN9 with EGFR may be the key factors that result in the reactivation of HER3 and acquired resistance to trastuzumab. We have shown that overexpression of PTPN9 resulted in HER3 dephosphorylation in trastuzumab acquired resistant cells. It remains to be established whether HER3 is a direct substrate of PTPN9 as immunoprecipitation experiments could not prove whether there is a direct interaction of PTPN9 and HER3 or whether their interaction is facilitated by EGFR and HER2. Previously, it was shown that trastuzumab disrupts ligand-independent HER2/HER3 interactions in HER2 positive breast cancer cells, which parallels HER3 dephosphorylation although the role of PTPN9 was not investigated [[26](#_ENREF_26)]. In addition, how PTPN9 interacts with EGFR, HER2 and HER3 receptors (either individually or their dimers complex) is likely to be complicated and is beyond the scope of this manuscript. Interaction between PTPN9 and all HER receptors are equally important to dephosphorylate HER receptors. However, in resistant cell lines EGFR and PTPN9 is lost with an unknown mechanism. It is likely that PTPN9 may be shuttling between EGFR, HER2 and HER3 as potential interacting partners during treatment pressure and this shuttling is lost in trastuzumab resistant cells or HER receptors might have different recycling/degradation cycle specially in trastuzumab resistant cells. Further detail investigation is required to delineate this paradigm. Alternatively, investigation in presence of other HER2 therapies that target different domain of HER2 (such as pertuzumab) or other HER receptors (e.g. EGFR or HER3 monoclonal antibody or other TKIs) may help to identify the mechanism of PTPN9 interactions with HER receptors. Furthermore, the molecular mechanism of the upregulation of PTPN9 in relation to trastuzumab treatment or other targeted therapies will need further investigation. A transcription factor like Foxo3a may be responsible since it has been reported that Akt inhibition induces the upregulation of multiple receptor tyrosine kinases including HER3, due to Foxo-dependent transcription [[27](#_ENREF_27)]. Considering that the effect is seen during acute treatment phase, post-translational regulation of PTPN9 could also be a possibility.

HER3 is important for tumourigenic signaling and thus a promising therapeutic target. Here, for the first time, we provide evidence that PTPN9 specifically regulates HER3 signaling in relation to trastuzumab treatment and resistance. An increased PTPN9 expression has been shown to impair oncogenic growth of HER2 over-expressed breast cancer cells and inhibits the growth and invasion of triple negative breast cancer cells by impairing the activation of STAT3/STAT5 through negatively regulating EGFR and HER2 receptors [[24](#_ENREF_24)]. However, we have not attempted to look at the STAT and ERK pathway. Since both these pathways play a crucial role in tumour progression, it will be important to explore both the STAT and ERK pathway in our experimental approach for complete inhibition of HER signalling in cancers. Various PTPs including PTPN12, PTPN13 have been implicated in tumour suppression [[4](#_ENREF_4), [28](#_ENREF_28)]. However, a detailed further investigation is needed to support the notion that PTPN9 has tumour suppressor activity. Since we found that EGFR is required for recruitment of HER3, it will be interesting to investigate the role of PTPN9 in head and neck cancer as well as lung cancer since EGFR is the critical driver of malignant progression in these malignancies. We also need to establish whether basal or post-treatment PTPN9 expression predicts sensitivity or resistance to trastuzumab treatment or any other HER2 therapies in both cell lines and organoid models, which will help to develop better treatment strategy for patients. Finally, our results identify PTPN9 as the PTP that could inhibit HER3 function by dephosphorylation and this could provide an important strategy in the design of novel cancer therapies.

In relation to clinical significance of PTPN9, we showed that there was a greater decrease of tumour size in patients with higher PTPN9 levels at day 21 after one dose of trastuzumab treatment in HER2 positive breast cancer patients. In addition, patients with low PTPN9 expression had poorer RFS and OS in a HER2 over-expressed breast cancer cohort, although only OS was statistically significant. The prognostic role of PTPN9 in OS was further validated in FinHER trial sample set, which was statistically significant in the patient group that included all patients with HER2-positive cancer and the non trastuzumab-treated subgroup but not in the trastuzumab-treated subgroup. It is important to note that the experimental arm patients were treated with adjuvant trastuzumab treatment in addition to chemotherapy and thus the mechanisms of resistance could be different from trastuzumab monotherapy. This was consistent with our data in the window study where PTPN9 expression was decreased after three weeks of trastuzumab monotherapy compared to the baseline but not after further trastuzumab treatment with neoadjuvant chemotherapy. We also did not see any statistical differences between the low PTPN9 and high PTPN9 expression in the distant disease-free survival (DDFS) in the overall group nor the subgroups of trastuzumab-treated and non trastuzumab-treated patients of FinHER trial patients [[10](#_ENREF_10)]. This could be due to improvement of DDFS as a result of adjuvant trastuzumab treatment in HER2 positive patients of FinHER trial plus a small number of patients analysed in the low PTPN9 groups. Thus, it will be important to further assess PTPN9 as a predictive biomarker for trastuzumab treatment and other anti-HER2 treatments in other independent set of large phase 3 randomised trials carried out in HER2 positive breast cancer patients. More investigation is also required to validate PTPN9 as a predictive biomarker for targeted therapies in various cancers. Targeting PTPN9 using an allosteric inhibitor of SHP2 could be a potential strategy to overcome drug resistance to trastuzumab, other targeted therapies and/or immunotherapy in clinic [[29](#_ENREF_29)]. This is yet to be demonstrated in preclinical models and could be done to validate PTPN9 as a potential target for therapy in cancers.

**Methods and materials**

**Organoid culture**: Breast cancer tumour and normal tissue was obtained after surgical resection from Queen Elizabeth Hospital, Birmingham (under the ethics of University of Birmingham Human Biomaterials Resource Centre (HBRC) reference 16-259). Tumour and normal tissue were processed for organoid formation according to published procedure [[7](#_ENREF_7), [30](#_ENREF_30)]. Briefly, after seceral washing in ADF^+++^ (Advanced DMEM/F12 containing 1x Glutamex, 10 mmol/L Hepes and penicillin/streptomycin, invitrogen) with Primocin. BC tissue were cut into small pieces and enzymatically digested using 1.5 mg/ml Collagenase (Sigma) for 1 hours at 37^◦^C in complete organoid medium. Tissue sections were sheared occasionally and after completion of digestion suspension was strained over a 70 µmol/L nylon membrane (Falcon) and centrifuge at 400g for 5 min. The resulting pellet was suspended in complete organoid medium containing ice -cold 10 mg/ml cold basement membrane (Matrigel Matrix, MG, Corning). 50 µl droplets of MG-cell suspension were plated on pre-warmed 24 -well suspension culture plates (Coster, Corning) for 20 min at 37^◦^C followed by the addition of 700 µl of BC organoid medium. The complete breast cancer organoid medium was prepared as published [[7](#_ENREF_7), [30](#_ENREF_30)]. Protein extract were made in RIPA buffer.

**Patient samples.** In the window of opportunity trial of HER2 positive breast cancer patients, both tumour size and response were assessed as previously described [[5](#_ENREF_5), [8](#_ENREF_8)]. Immunohistochemical evaluation was performed for HER2, ERα, PgR, and Ki67, as described [[31](#_ENREF_31)].

The trial was conducted at UOM Patologia Mammaria-Az. Instituti Ospitalieri di Cremona with appropriate local ethical approval (Protocol CE-21392/2012). The TMA slides from a cohort of HER2 positive breast cancer patients were provided by Oxford Radcliffe Biobank after an internal application and reviewed by the Scientific and Ethical Review Committee. The use of these TMA slides complies with the Human Tissue Act 2004 (UK). The FinHER trial results were previously published [[10](#_ENREF_10)] and the current study was done under the study proposal approved by the Helsinki University Central Hospital Ethics Committee (331/E6/07, 17 Oct 2007). The trial patients provided written informed consent for the use of their tumour tissue material for the FinHER trial-related research.

**PTPN9 IHC scoring.** PTPN9 expression level was scored semi-quantitatively based on staining intensity and distribution using the immunoreactive score (IRS) as described [[9](#_ENREF_9)]. The low expression or no expression of PTPN9 in tumour samples is determined by IRS scoring, using IRS less than 4 21, which will consist of mainly tumours with either no or weak staining in majority of the tumour cells.

**Statistical Analysis.** Prism version 5 (GraphPad) was used for statistical analysis and *P*-values are depicted. The correlation of the intensities of pHER3 blots with that of PTPN9 was performed using Pearson correlation test. Associations between co-variables and PTPN9 expression were tested by Fisher’s exact test. This and the multivariate Cox proportional hazards modeling and the Kaplan-Meier survival curves analysis were done in R statistical environment (v.2.14.1) (R package: survival v2.36-14). For FinHER trial OS and RFS were assessed using Log-rank test. The tests of statistical significance were two-sided and *P*-values less than 0.05 were considered statistically significant.

**Cell culture, transfection and reagents.** SKBR3, MDA-MB-453 and MDA-MB-361 cells were cultured in DMEM supplemented with 10% FCS, 1% Penicillin/Streptomycin. BT474 was grown in RPMI supplemented with 10% FCS, 1% Penicillin/Streptomycin. Both SKBR3 and BT474 trastuzumab resistant cell lines were generated by continuously growing them in presence of 40 µg/ml of trastuzumab as previously described (e). PTPN9 specific siRNAs were purchased from Ambion (oligo1: ID-s11522 and oligo2: ID-s11521) and from Thermo Scientific Dharmacon (oligo3: cat # J-008832-05 and oligo4:cat no# J-008832-07). siRNAs were transfected into cells using Dermafect1 (Thermo fisher) reagent according to manufacturer’s instructions. PTPN9 overexpressing plasmids (WT, C515S and D470A) were kindly provided by Dr. Charles Cho (Genentec) [[6](#_ENREF_6)]. Plasmid DNAs were transfected into cells using Lipofectamine (Invitrogen) and selected with G418 (Sigma). Trastuzumab was purchased from the pharmacy department of Oxford Radcliffe Hospitals, NHS trust.

**Immunobloting, Immunoprecipitation and antibodies**. Cell lysates were prepared in either RIPA buffer for western blotting or IP lysis buffer (20mmol/L Tris-HCl, pH7.4, 150mmol/L NaCl, 1.5mmol/L MgCl2, 5mmol/L EDTA, 1% TritonX-100, 10% glycerol containing protease inhibitor cocktail [Roche], phosphatse inhibitor cocktail 2 and 3 [Sigma]) for immunoprecipitation. Cells were lysed for 30 min on ice and supernatant containing protein extracts were collected by centrifugation at 13,000 rpm for 10 min. 30-60 µg of proteins were separated on SDS-PAGE (Invitrogen) and transferred to Hybond-C membranes (Amersham Biosciences). Standard procedures were applied for western blotting. For immunoprecipitations, protein extracts were incubated overnight at 4°C with the respective antibody. 30 µl of a 50% slurry of either TrueBlot anti mouse IgG or anti rabbit IgG (Rockland) were added to the samples and incubated at 4°C for 90 min. The beads were then washed in IP buffer 3 times and dissolved in 25µl of 2X SDS buffer. The following antibodies were used: mouse anti PTPN9 (Santacruz), rabbit anti PTPN9 (Sigma), rabbit HER2, pHER2 (Y1221), pHER3 (Y1289), pHER3 (Y1197), Akt, pAkt (S473), EGFR, Actin (all from Cell signalling), mouse HER3 (abcam), rabbit pEGFR (Y1173) (Santacruz).

**Immunostaining**. Cells were grown on coverslips for 24 hours and treated with trastuzumab for 4 hours. After 3 washes with PBS, cells were then fixed with 4% paraformaldehyde in PBS for 15 min and washed with PBS. The fixed cells were permeabilized with 0.15% Triton X-100 for 15 min at RT and blocked in 5% BSA for 1-2 hr. The mouse anti PTPN9 antibody was used at 1:50 and rabbit anti HER3 antibody was used at 1:200 for 2-3 hr at RT in 1%BSA. Following 3 washes with PBS, cover slips were incubated with either anti mouse Alexa-Flour 488 and anti- rabbit Alexa-Flour 546 (Invitrogen). Cover slips were mounted with Fluoromount-G (Southern biotec) containing DAPI. Images were obtained by confocal laser scanning microscopy (Zeiss LSM510) using a 488 nm Argon II laser (green fluorescence), a 561 nm DPSS561-10 laser (red fluorescence) and a 405 nm Diode.

**Immunohistochemistry.** The TMA slides were deparaffinized in 2 changes of citroclear solution (5 minutes each) and then hydrated in 2 changes of 100% ethanol (5 minutes each). This was followed by 70% and 50% ethanol for 5 min each and then rinsed in distilled water. Antigen retrieval was done by heating the slides in citrate buffer (10mmol/L citric acid, 0.05% Tween 20, pH 6.0) for 2 min at 125 °C and 10 min at 85 °C. Following antigen retrieval, the sections were rinsed with PBS before being blocked and stained with primary antibody mouse anti PTPN9 diluted in RPMI medium overnight at 4°C. After rinsing with PBS twice, the sections were incubated with peroxidase-linked anti-rabbit Ig (ImmPRESS) for 30 minutes at room temperature. Then the slides were rinsed with PBS twice and incubated with 3,3’-Diaminobenzidine solution for 5 minutes. A counterstaining was performed by incubating the slides in hematoxylin QS solution (Vector Laboratories) for 30 seconds. Sections were mounted using an aqueous mounting solution (Aquatex).

**References**

1. Gijsen M, King P, Perera T, Parker PJ, Harris AL, Larijani B, et al. HER2 phosphorylation is maintained by a PKB negative feedback loop in response to anti-HER2 herceptin in breast cancer. PLoS Biol. 2010;8(12):e1000563.

2. Yuan T, Wang Y, Zhao ZJ, Gu H. Protein-tyrosine phosphatase PTPN9 negatively regulates ErbB2 and epidermal growth factor receptor signaling in breast cancer cells. J Biol Chem. 2010;285(20):14861-70.

3. Flint AJ, Tiganis T, Barford D, Tonks NK. Development of "substrate-trapping" mutants to identify physiological substrates of protein tyrosine phosphatases. Proc Natl Acad Sci U S A. 1997;94(5):1680-5.

4. Zhu JH, Chen R, Yi W, Cantin GT, Fearns C, Yang Y, et al. Protein tyrosine phosphatase PTPN13 negatively regulates Her2/ErbB2 malignant signaling. Oncogene. 2008;27(18):2525-31.

5. Feldinger K, Generali D, Kramer-Marek G, Gijsen M, Ng TB, Wong JH, et al. ADAM10 mediates trastuzumab resistance and is correlated with survival in HER2 positive breast cancer. Oncotarget. 2014;5(16):6633-46.

6. Cho CY, Koo SH, Wang Y, Callaway S, Hedrick S, Mak PA, et al. Identification of the tyrosine phosphatase PTP-MEG2 as an antagonist of hepatic insulin signaling. Cell Metab. 2006;3(5):367-78.

7. Arshad M, Azad A, Chan PYK, Vigneswara V, Feldinger K, Nafi SNM, et al. Neratinib could be effective as monotherapy or in combination with trastuzumab in HER2-low breast cancer cells and organoid models. Br J Cancer. 2024;130(12):1990-2002.

8. Mohd Nafi SN, Generali D, Kramer-Marek G, Gijsen M, Strina C, Cappelletti M, et al. Nuclear HER4 mediates acquired resistance to trastuzumab and is associated with poor outcome in HER2 positive breast cancer. Oncotarget. 2014;5(15):5934-49.

9. Nagata Y, Lan KH, Zhou X, Tan M, Esteva FJ, Sahin AA, et al. PTEN activation contributes to tumor inhibition by trastuzumab, and loss of PTEN predicts trastuzumab resistance in patients. Cancer Cell. 2004;6(2):117-27.

10. Joensuu H, Kellokumpu-Lehtinen PL, Bono P, Alanko T, Kataja V, Asola R, et al. Adjuvant docetaxel or vinorelbine with or without trastuzumab for breast cancer. N Engl J Med. 2006;354(8):809-20.

11. Linggi B, Carpenter G. ErbB receptors: new insights on mechanisms and biology. Trends Cell Biol. 2006;16(12):649-56.

12. Slamon DJ, Clark GM, Wong SG, Levin WJ, Ullrich A, McGuire WL. Human breast cancer: correlation of relapse and survival with amplification of the HER-2/neu oncogene. Science. 1987;235(4785):177-82.

13. Yarden Y, Pines G. The ERBB network: at last, cancer therapy meets systems biology. Nat Rev Cancer. 2012;12(8):553-63.

14. Yarden Y, Sliwkowski MX. Untangling the ErbB signalling network. Nat Rev Mol Cell Biol. 2001;2(2):127-37.

15. Slamon DJ, Leyland-Jones B, Shak S, Fuchs H, Paton V, Bajamonde A, et al. Use of chemotherapy plus a monoclonal antibody against HER2 for metastatic breast cancer that overexpresses HER2. N Engl J Med. 2001;344(11):783-92.

16. Wynn CS, Tang SC. Anti-HER2 therapy in metastatic breast cancer: many choices and future directions. Cancer Metastasis Rev. 2022;41(1):193-209.

17. de Melo Gagliato D, Jardim DL, Marchesi MS, Hortobagyi GN. Mechanisms of resistance and sensitivity to anti-HER2 therapies in HER2+ breast cancer. Oncotarget. 2016;7(39):64431-46.

18. Olayioye MA, Neve RM, Lane HA, Hynes NE. The ErbB signaling network: receptor heterodimerization in development and cancer. EMBO J. 2000;19(13):3159-67.

19. Sergina NV, Rausch M, Wang D, Blair J, Hann B, Shokat KM, et al. Escape from HER-family tyrosine kinase inhibitor therapy by the kinase-inactive HER3. Nature. 2007;445(7126):437-41.

20. Tonks NK. Protein tyrosine phosphatases: from genes, to function, to disease. Nat Rev Mol Cell Biol. 2006;7(11):833-46.

21. Keilhack H, Tenev T, Nyakatura E, Godovac-Zimmermann J, Nielsen L, Seedorf K, et al. Phosphotyrosine 1173 mediates binding of the protein-tyrosine phosphatase SHP-1 to the epidermal growth factor receptor and attenuation of receptor signaling. J Biol Chem. 1998;273(38):24839-46.

22. Kuang W, Wang X, Ding J, Li J, Ji M, Chen W, et al. PTPN2, A Key Predictor of Prognosis for Pancreatic Adenocarcinoma, Significantly Regulates Cell Cycles, Apoptosis, and Metastasis. Front Immunol. 2022;13:805311.

23. Sun T, Aceto N, Meerbrey KL, Kessler JD, Zhou C, Migliaccio I, et al. Activation of multiple proto-oncogenic tyrosine kinases in breast cancer via loss of the PTPN12 phosphatase. Cell. 2011;144(5):703-18.

24. Yuan T, Wang Y, Zhao ZJ, Gu H. Protein-tyrosine phosphatase PTPN9 negatively regulates ErbB2 and epidermal growth factor receptor signaling in breast cancer cells. J Biol Chem. 2010;285(20):14861-70.

25. Kong A, Calleja V, Leboucher P, Harris A, Parker PJ, Larijani B. HER2 oncogenic function escapes EGFR tyrosine kinase inhibitors via activation of alternative HER receptors in breast cancer cells. PLoS One. 2008;3(8):e2881.

26. Junttila TT, Akita RW, Parsons K, Fields C, Lewis Phillips GD, Friedman LS, et al. Ligand-independent HER2/HER3/PI3K complex is disrupted by trastuzumab and is effectively inhibited by the PI3K inhibitor GDC-0941. Cancer Cell. 2009;15(5):429-40.

27. Chandarlapaty S, Sawai A, Scaltriti M, Rodrik-Outmezguine V, Grbovic-Huezo O, Serra V, et al. AKT inhibition relieves feedback suppression of receptor tyrosine kinase expression and activity. Cancer Cell. 2011;19(1):58-71.

28. Sun T, Aceto N, Meerbrey KL, Kessler JD, Zhou C, Migliaccio I, et al. Activation of multiple proto-oncogenic tyrosine kinases in breast cancer via loss of the PTPN12 phosphatase. Cell. 2011;144(5):703-18.

29. Zhao M, Shuai W, Su Z, Xu P, Wang A, Sun Q, et al. Protein tyrosine phosphatases: emerging role in cancer therapy resistance. Cancer Commun (Lond). 2024;44(6):637-53.

30. Sachs N, de Ligt J, Kopper O, Gogola E, Bounova G, Weeber F, et al. A Living Biobank of Breast Cancer Organoids Captures Disease Heterogeneity. Cell. 2018;172(1-2):373-86 e10.

31. Generali D, Buffa FM, Berruti A, Brizzi MP, Campo L, Bonardi S, et al. Phosphorylated ERalpha, HIF-1alpha, and MAPK signaling as predictors of primary endocrine treatment response and resistance in patients with breast cancer. J Clin Oncol. 2009;27(2):227-34.
